# The role of bone marrow mononuclear cell-conditioned medium in the proliferation and migration of human dermal fibroblasts

**DOI:** 10.1186/s11658-017-0055-z

**Published:** 2017-12-19

**Authors:** Yolanda Menéndez-Menéndez, Jesús Otero-Hernández, Jose Antonio Vega, Marcos Pérez-Basterrechea, Silvia Pérez-López, María Álvarez-Viejo, Amaia Ferrero-Gutiérrez

**Affiliations:** 1 Unidad de Coordinación de Trasplantes, Terapia Celular y Medicina Regenerativa, Hospital Universitario Central de Asturias, Oviedo, Spain; 2 Departamento de Morfología y Biología Celular, Universidad de Oviedo, Oviedo, Spain; 3 Facultad de Ciencias de la Salud, Universidad Autónoma de Chile, Temuco, Chile

**Keywords:** Bone marrow mononuclear cells, Cell migration and proliferation, Human dermal fibroblasts, Paracrine interactions, Wound repair

## Abstract

**Background:**

Several recent studies have demonstrated the great potential of bone marrow cells in regenerative medicine, not only for their ability to differentiate to match a damaged cell type, but also because they synthesize and release various growth factors and cytokines.

We examined the effect of bone marrow cell-conditioned medium in the healing process, especially in terms of fibroblast proliferation and migration.

**Methods:**

These in vitro studies consisted of co-culture (without direct contact) of dermal fibroblasts with mononuclear bone marrow cells and the use of conditioned medium obtained from these cultures in a scratch wound model.

**Results:**

Mononuclear cells were found to increase the proliferation of fibroblasts, and the conditioned medium showed a stimulatory effect on the migration of fibroblasts.

**Conclusion:**

When considered together with the observed increase in growth factor levels in conditioned medium, it appears that these cells act through a paracrine mechanism.

## Background

Wound healing is a complex process that involves various biological systems interacting through cell-to-cell contacts and diffusible factors [1, 2]. It develops in three sequential overlapping steps: inflammation, cell proliferation and cell maturation. This process is regulated by an intricate signaling network involving numerous growth factors, cytokines and chemokines. Members of the families of epidermal growth factors (EGF), beta transforming growth factors (TGF-β), fibroblast growth factors (FGF) and platelet-derived growth factors (PDGF) are among the proteins that play essential roles in this network [3]. Thus, successful wound healing depends on the timely and optimal functioning of many diverse processes that lead to the generation of new tissue. The failure of any of these steps results in a chronic wound.

Chronic ischemic wounds are essentially hypoxic. In the literature, hypoxia is generally viewed as angiogenic. This is true if the hypoxia is acute and mild to modest in magnitude. Extreme near-anoxic hypoxia, as commonly noted in problem wounds, is not compatible with tissue repair [4].

The conventional treatments for chronic wounds include debridement of necrotic tissue, minimizing of bacterial load, pressure offloading, negative-pressure therapy, biological dressing, skin grafting, and reconstructive tissue flaps [5]. However, despite recent advances in wound management, a high percentage become chronic wounds, and up to 50% of chronic wounds still fail to heal [6, 7].

Other therapeutic strategies have been explored for the treatment of chronic wounds, including cell therapy using various kinds of adult stem cells, especially those from the bone marrow (BM) [8–11]. Bone marrow mononuclear cells (BM-MNCs) are one subtype that includes hematopoietic stem cells (HSC), mesenchymal stem cells (MSC) and endothelial progenitors [12]. BM cells have been used for treating chronic wounds in humans [13–19]. Autologous injections of BM-MNCs have proven highly effective in the treatment of critical limb ischemia patients [20] and grade IV pressure ulcers [21].

Fibroblasts play a key role in wound healing and tissue repair because they are the main source of growth factors (GFs), adhesion molecules and the extracellular matrix (ECM) component required for tissue formation and angiogenesis [22, 23]. Thus, the proliferation and subsequent migration of fibroblasts after a wound occurs are limiting for wound repair and healing. Both steps are regulated by a complex signaling network of diffusible molecules that includes GFs, cytokines and chemokines [24].

In vitro wound models have shown that MSCs induce the proliferation of fibroblasts without cell-to-cell contact [25, 26], suggesting that soluble factors released to the medium from MSCs are responsible for this effect, acting in a paracrine and/or autocrine manner [26].

To investigate whether BM-MNCs have similar effects to those of MSCs in chronic wound healing and whether they act through similar mechanisms, we examined the in vitro effects of a BM-MNC-conditioned medium on human dermal fibroblast proliferation and migration. The aim was to better understand the cellular and molecular mechanisms underlying the role of BM-MNCs in wound repair.

## Methods

The study was carried out at the Hospital Universitario Central de Asturias (HUCA), Oviedo (Spain) in the Laboratory of Unidad de Coordinación de Trasplantes, Terapia Celular y Medicina Regenerativa. It was conducted in agreement with the ethical standards upheld by Spanish and European laws.

BM-MNCs were obtained from 20 patients undergoing cell therapy. Each signed a detailed informed consent form, and the study was authorized by the HUCA Ethical Committee (Clinical Trial NCT 01572376). In addition, human dermal fibroblasts were obtained from 5 multi-organ donors aged 20–30 years, whose families signed a detailed document authorizing the use of the organs and tissues of donor. No additional committee consent was required for these experiments.

### Isolation and culture of human dermal fibroblasts

Adult human dermal fibroblasts were obtained as previously described [27] with some modifications. Briefly, skin biopsy specimens were obtained using an 8-mm punch, then the pieces were divided into small fragments and digested with 2 mg/ml collagenase I (Sigma-Aldrich) in Dulbecco’s modified Eagle’s medium (DMEM, Gibco Invitrogen) supplemented with 2% antibiotic–antimycotic solution (200 U/ml penicillin, 200 μg/ml streptomycin, 0.5 μg/ml amphotericin B; Life Technologies). The suspension was shaken for approximately 3 h at 37 °C until fragments were completely digested. After filtering through a 40 μm cell strainer (BD), and centrifugation at 400 g for 10 min at 20 °C, the cells were seeded in 25 cm^2^ polystyrene flasks at 1 × 10^5^ cells/cm^2^ in a culture medium consisting of DMEM supplemented with 10% fetal bovine serum (FBS; Gibco, Invitrogen), 1% L-glutamine and 1% antibiotic solution (100 U/ml penicillin and 100 μg/ml streptomycin). Cells were cultured in normoxia (37 °C, 5% CO_2_, 21% O_2_). After 48 h, the medium was replaced and unattached cells were removed. The medium was thereafter replenished every 2 to 3 days, and when cells reached a confluence of 90%, they were digested with trypsin (0.25% *w*/*v*). The fibroblasts were used for the experiments after 4–7 passages.

### Proliferation of human dermal fibroblasts

Human dermal fibroblasts were seeded at 1 × 10^4^ cells per well (in 6-well plates) in culture medium and left to grow for 9 days (seeding counted as day 0 in the overall experimental timeline). Each day, fibroblasts were trypsinized and counted in a Neubauer hemocytometer. This experiment was performed both in a standard controlled atmosphere (37 °C, 21% O_2_) and a hypoxic atmosphere (37 °C, 5.5–12% O_2_). Hypoxic conditions were generated using a GENbag Microaer (BioMerieux) cell incubation system, according to the manufacturer’s instructions.

### Isolation of BM-MNCs

BM-MNCs were obtained as reported earlier [21]. In brief, BM was harvested by posterosuperior iliac crest aspiration under topical anesthesia (mepivacaine 2%, B. Braun) and MNC isolated from the aspirate on a Ficoll density gradient by centrifuging for 25 min at 400 g and 20 °C. The cells were finally re-suspended in culture medium (DMEM supplemented with 10% fetal bovine serum, 1% L-glutamine and 1% antibiotic solution) and counted in a Neubauer hemocytometer.

In a previous study, we quantified the MSC population in BM samples using flow cytometry to detect the CD271 antigen, demonstrating a low percentage as well as a high variability of CD271_CD45_ cells (range, 0.0017 to 0.0201%) among subjects [28].

### Human dermal fibroblast/BM-MNC co-cultures

**Fig. 1:**
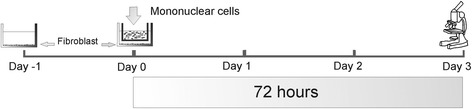
Fibroblast proliferation assay. 1 × 10^4^ human dermal fibroblasts were cultured for one day (day −1) prior to their co-culture (day 0) with 5 × 10^6^ BM-MNCs in inserts. Fibroblasts were counted after 72 h (day 3) of co-culture

Human dermal fibroblasts were co-cultured with BM-MNCs in cell culture inserts with 40-μm pores, which allowed for paracrine interactions without direct cell-to-cell contact. For these experiments, the fibroblasts were seeded at 1 × 10^3^ cells/cm^2^ in 6-well plates and cultured for one day (day −1) prior to co-culture (day 0) with 5 × 10^6^ BM-MNCs in the inserts. The co-culture was kept in culture medium. After co-culture (3 days), the culture medium (hereafter referred to as conditioned medium) was recovered and cell debris detached by filtration (0.22-μm filter). After trypsinizing, the fibroblasts were counted in a Neubauer hemocytometer (see Fig. 1). These experiments were performed in both standard and hypoxic atmosphere.

### Culturing human dermal fibroblasts in the MNC-conditioned medium

Fibroblasts were seeded at 1 × 10^3^ cells/cm^2^ in 6-well plates and cultured for one day (day −1) before the addition of 1.5 ml of the conditioned medium (day 0). After 3 days of culturing in this medium (day 3), the fibroblasts were trypsinized and counted in a Neubauer hemocytometer. These experiments were performed in a standard or hypoxic atmosphere (to simulate the wound local microenvironment).

### Scratch wound model

The cell migration assay was performed using a modification of the protocol described by Liang et al. [29]. Fibroblasts were seeded at 5 × 10^3^ cell/cm^2^ per well in 24-well plates and cultured in a standard controlled atmosphere until the formation of a confluent monolayer. Confluent monolayers were then scored with a sterile pipette tip to leave a scratch approximately 0.5–0.6 mm wide. The culture medium was then immediately removed and replaced with the versions of the conditioned medium obtained in both standard and hypoxic atmospheres.

The fibroblasts were kept in both controlled atmospheres and wound closure was monitored by taking digitized images of four randomly selected culture fields immediately after scratching and every 12 h until the wound had closed (100% confluent). The images were captured with an inverted microscope (Olympus IX71) and digital camera (Olympus DP71) and analyzed with the Image J software (version 1.45) to measure the width of the scratch.

### Measurement of GFs in the conditioned medium

The presence of VEGF, FGF, EGF and PDGF in the conditioned medium was determined using enzyme-linked immunosorbent assay (ELISA) kits following the manufacturer’s instructions (RayBiotech, Norcross,). Fluorescence was measured at an emission wavelength of 450 nm using a microplate absorbance reader (BioRad). The concentrations of the growth factors were calculated using standard solutions as reference.

### Statistical analysis

All statistical tests were performed using the software package Graphpad InStat 3 for Windows. Data were compared using unpaired two-tailed one-way analysis of variance (ANOVA) or Student’s t test. Data are provided as means ± SEM. Significance was set at *p* < 0.05. Figures only indicate significant differences relevant to the discussion of the data.

## Results

### Human dermal fibroblast growth

**Fig. 2:**
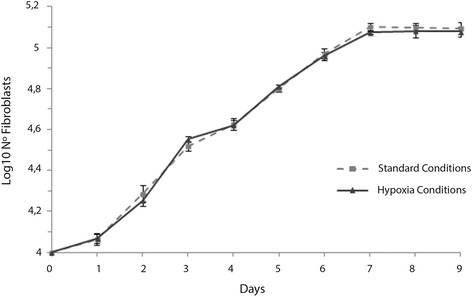
Human dermal fibroblast growth assay. 1 × 10^4^ fibroblasts were seeded (day 0) and left to grow for 9 days in standard culture or hypoxic conditions. Fibroblasts were counted each day. Results are expressed as means ± SEM (*n* = 5 to 9)

As a preliminary step, we established the rate of fibroblast growth over a 9-day period in conditions of both standard culture and hypoxia. Wells seeded with 10,000 fibroblasts (10% confluence) showed exponential growth for the first 7 days in both environmental conditions (Fig. 2) After 3 days of culture, the number of fibroblasts had more than tripled: 33,000 ± 1700 and 45% confluence and 35,500 ± 1100 and 50% confluence respectively for the standard culture and hypoxia conditions. By day 7, they reached the maximal confluence (100%) with counts of 120,000 fibroblasts recorded in both environmental conditions. Thereafter, confluence values and cell counts remained stable until day 9 (Fig. 2). These results indicate that the growth and confluence of cultured human fibroblasts was not influenced by the relative hypoxia. Based on these observations, the subsequent proliferation experiments were focused on the exponential phase of growth (first 3 days).

### BM-MNCs induce human dermal fibroblast proliferation in vitro

**Fig. 3:**
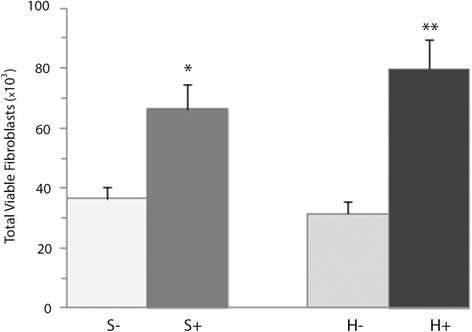
Effects of BM-MNCs on the proliferation of fibroblasts after 3 days of co-culture in standard (S+) and hypoxic atmospheres (H+). The results are expressed as means ± SEM (*n* = 20 standard culture, *n* = 17 hypoxic). *p < 0.05 compared with control in the standard atmosphere (S-). **p < 0.001 compared with the control in the hypoxic atmosphere (H-)

To explore the paracrine effects of BM-MNCs on fibroblast proliferation, we co-cultured human dermal fibroblasts with human BM-MNCs grown in inserts without direct cell contact. In the standard controlled atmosphere, fibroblast proliferation was much enhanced by the presence of BM-MNCs. After 3 days of co-culture, the number of fibroblasts almost doubled in presence of BM-MNCs (66,500 ± 8000) compared with cultures without BM-MNCs (36,600 ± 4000; *p* < 0.05; Fig. 3). This increase was even more evident in the hypoxic controlled atmosphere, where counts of 80,000 ± 10,000 were recorded vs. 31,400 ± 4000 for controls (p < 0.05; Fig. 3). Because no cell-to-cell contact existed, the observed effects could be due to a paracrine loop between fibroblasts and BM-MNCs.

### A BM-MNC-conditioned medium enhances the in vitro proliferation of human dermal fibroblasts, mediated by soluble growth factors

**Fig. 4:**
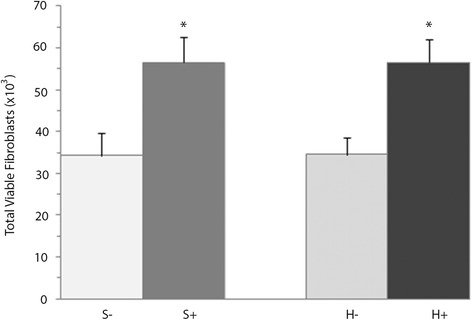
Effects of the BM-MNC conditioned-medium on the proliferation of fibroblasts after 3 days in standard (S+) or hypoxic atmosphere (H+). The results are expressed as means ± SEM (*n* = 19 standard culture, *n* = 15 hypoxic). *p < 0.05 compared with control in the same atmosphere (S- or H-)

To corroborate the paracrine effects of BM-MNCs on dermal fibroblast proliferation, fibroblasts were cultured in the conditioned medium. After 3 days of culture, fibroblast counts were significantly higher (p < 0.05) than in the controls, both under standard conditions (56,500 ± 6200 vs. 34,300 ± 5400) and hypoxia (56,500 ± 5500 vs. 34,600 ± 4100; Fig. 4).

**Fig. 5:**
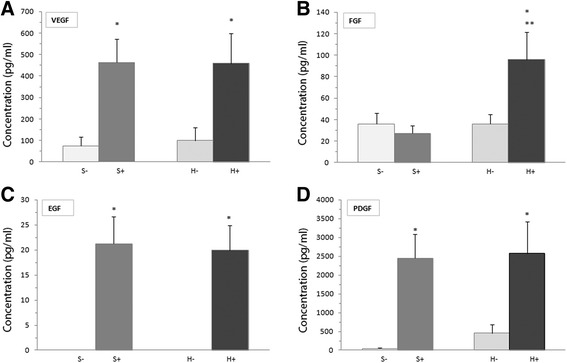
Growth factors detected in the conditioned medium in standard (S+) and hypoxic (H+) atmospheres. Concentrations of VEGF (**a**), FGF (**b**), EGF (**c**) and PDGF (**d**) were measured using ELISA. The results are expressed as means ± SEM (*n* = 19 standard culture, *n* = 15 hypoxic). *p < 0.01 compared with the control in the same atmosphere (S- or H-). **p < 0.05 compared with S+

We then investigated the presence of some GFs in the conditioned medium. The conditioned medium contained significantly higher concentrations of VEGF, EGF and PDGF, but not of FGF, than the control medium obtained from cultured fibroblasts alone (Fig. 5). In hypoxia conditions, the concentrations of all GFs examined were significantly higher in the conditioned medium than in the control one (Fig. 5).

### Effect of the conditioned medium on human dermal fibroblast migration

**Fig. 6:**
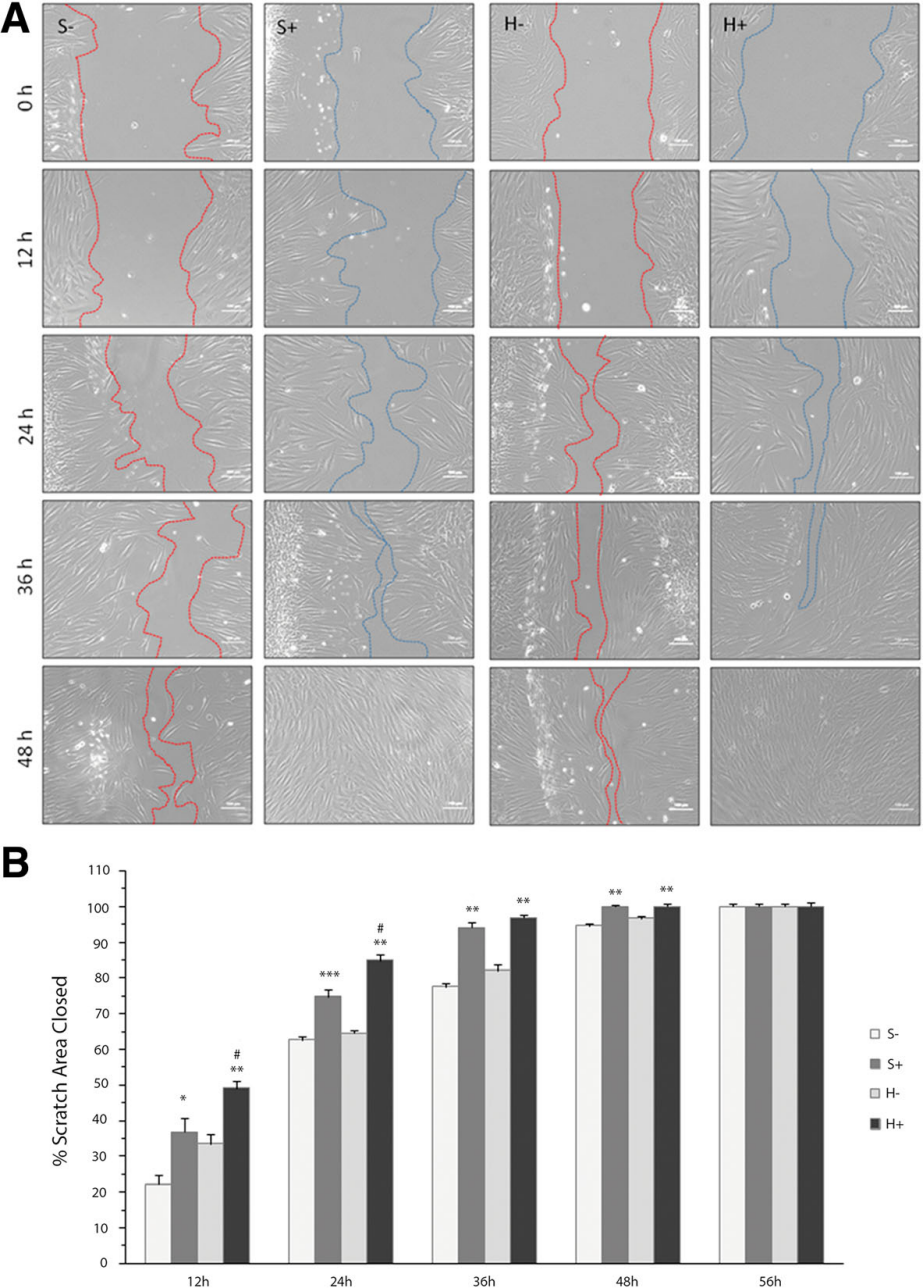
Effects of the BM-MNC-conditioned medium on fibroblast migration in the wound scratch assay. **a** – A confluent monolayer of fibroblasts was scratched and the conditioned medium obtained in a standard (S+) or hypoxic (H+) atmosphere was then added. Images were captured at 0, 12, 24, 36, 48 and 56 h (data not shown) and compared with their control in the same atmosphere (S- or H-). Scratch closure was measured as the width of each scratch using Image J software (version 1.45). **b** – Each time point was normalized to the image perimeter on day 0 and reported as percent closure. The results are expressed as means ± SEM (*n* = 6). *p < 0.05 compared with the control in the same atmosphere (S- or H-). **p < 0.001 compared with the control in the same atmosphere (S- or H-). ***p < 0.01 compared with the control in the standard atmosphere (S-). ^#^ *p* < 0.01 compared with S+

Finally, we investigated whether the conditioned medium affects fibroblast migration in a series of in vitro wound closure migration experiments. In the standard cultures, the conditioned medium induced a significantly shorter timeframe of fibroblast migration compared to the controls at the time points 12, 24, 36 and 48 h (Fig. 6a, S-, S+). It should be stressed that early on (≤ 24 h) in conditions of hypoxia, the migration of fibroblasts was potentiated by the hypoxia while by 36 h of culture and later, no difference in migration was detected between the two conditions tested (Fig. 6b). After 48 h, wound closure was complete (100% confluence) in the fibroblast cultures exposed to the conditioned medium compared with the untreated ones, which showed about 90% closure then, and 100% closure after 56 h (Fig. 6b). The results in hypoxic conditions were almost identical (Fig. 6a, H-, H+, and b). For the hypoxic controls, 100% wound closure was observed in conditioned medium-exposed fibroblasts after 48 h versus 56 h.

## Discussion

Chronic wounds are a common clinical and socio-sanitary problem: a high percentage of patients do not respond to conventional treatment [30]. There has been a rapid increase in the development of cost-effective wound healing technologies [31] Each therapeutic safety strategy able to reduce the wound healing time and prevent wound chronicity is welcome [32].

Based on our previous promising results using BM-MNCs to treat pressure ulcers in patients with spinal cord injury [21], we designed this study to look at the effects of BM-MNCs on the proliferation and migration of human dermal fibroblasts, which have an important role in synthesizing ECM components and releasing biologically active molecules [23].

To simulate the wound local microenvironment, assays were performed with a reduced amount of oxygen (hypoxic; 5.5–12% O_2_) and the results compared with those obtained in standard culture conditions (21% O_2_). Hypoxia had no effect on fibroblast proliferation, but the presence of BM-MNCs and hypoxic conditions potentiated fibroblast proliferation. This is in line with previous experiments demonstrating enhanced proliferation of cutaneous fibroblasts by BM-MSCs and adipose MSCs in hypoxic conditions [33, 34]. However, the effects of hypoxia on short- or long-term MSC proliferation vary according to oxygen concentration, culture conditions, MSC seeding density and MSC source [35–38].

To analyze the effects of BM-MNCs on human dermal fibroblast proliferation, we used inserts that prevent direct contact between the cell types. Fibroblast proliferation was significantly enhanced when co-cultured with BM-MNCs and after exposure to BM-MNC-conditioned medium, in both standard and hypoxic culture conditions. Because the results concurred in both experiments, it seems evident that interactions between BM-MNCs and fibroblasts occur through paracrine mechanisms. These findings concur with those from studies that proposed paracrinia as the main mechanism between adult stem cells and other cell types responsible for cutaneous wound repair [33, 39–41].

The second main goal of this research was to analyze the effects of BM-MNCs on human dermal fibroblast migration, because of its importance in wound healing [42]. Disruption of cell-to-cell contact by a wound induces an increase in GF concentrations at the wound margins. GFs are responsible not only for cell proliferation but also for migration [9].

Here, we used a straightforward in vitro scratch assay to examine human dermal fibroblast migration [43] and observed that the conditioned medium from BM-MNC/fibroblast co-cultures accelerates wound closure (48 h in conditioned vs. 56 h medium in control medium), enhancing cell migration independently of the oxygen concentrations. Similar findings were obtained using a conditioned medium of human BM-MSCs [44]. Although this migratory effect of the conditioned medium was not reported earlier, it can be regarded as similar to the migratory effects induced by these cells in murine macrophages, keratinocytes and endothelial cells [34]. Further research is needed to elucidate the possible functions of the BM-MNC/fibroblast-conditioned medium in cutaneous cells other than fibroblasts.

The complex process of wound healing involves the coordinated actions of several cytokines and GFs, whose coordinated functions regulate the different steps of the tissue repair process [45]. Our findings indicate that the co-culture of fibroblasts and BM-MNCs induces the secretion of several GFs involved in wound healing, which may have a direct effect on cell proliferation and migration [46, 47]. In this study, we analyzed the possible role of some GFs that presumably mediate the relationships between BM-MNCs and fibroblasts by detecting them in the conditioned medium. Increased amounts of VEGF, EGF and PDGF were detected in the medium derived from the BM-MNC/fibroblast co-cultures relative to the controls under both standard and hypoxic culture conditions. By contrast, FGF secretion was significantly higher only in the hypoxic cultures, suggesting that whereas VEGF, EGF and PDGF are critical for fibroblast proliferation and migration, FGF are not, at least in conditions of normoxia.

Nevertheless, the significant FGF increase in the BM-MNC/fibroblast-conditioned medium in conditions of hypoxia could be related to the fibroblast migratory behavior, because we observed that under hypoxic conditions and for short times (≤ 24 h), the ratio (%) of wound closure was remarkably higher for fibroblasts exposed to a conditioned medium compared to those cultured in standard conditions.

On the other hand, although no information exists on BM-MNCs, our results are consistent with previous results using mouse adipose MSCs [14] or BM-MSCs [34]. In a previous study, we quantified the MSC population in BM samples using flow cytometry to detect the CD271 antigen, demonstrating a low percentage of MSCs, discarding the possibility that there are plenty of MSCs in the isolated BM-MNCs [28].

## Conclusion

These results support the idea that BM-MNCs are suitable candidates for use in cell therapy for wound treatment because they potentiate human dermal fibroblast proliferation and migration. Compared to MSCs, these cells have the benefit that they can be obtained in about 3 to 4 h in sufficient numbers to preclude the need for culture. This avoids excessive manipulation, reducing the risk of contamination.

Moreover, because the beneficial effects of BM-MNCs on cutaneous fibroblasts are mediated, at least in part, by released molecules, i.e., GFs, the possibility to store, easily transport and repeatedly use these molecules opens a new possibility in wound treatment, especially for chronic wounds.

## Abbreviations

BM: Bone marrow
ECM: Extracellular matrix
EGF: Epidermal growth factors
FGF: Fibroblast growth factors
HSC: Hematopoietic stem cells
MNC: Mononuclear cells
MSC: Mesenchymal stem cells
PDGF: Platelet-derived growth factor
TGF-β: Beta transforming growth factors
